# Editorial: Application of natural medicinal products in preventing and ameliorating aging-caused cognitive impairment

**DOI:** 10.3389/fphar.2022.1059398

**Published:** 2022-11-03

**Authors:** Hongyu Li, Guangyu Xu, Wenhe Zhu, Guangxin Yuan

**Affiliations:** ^1^ School of Pharmacy, Beihua University, Jilin, China; ^2^ School of Pharmacy, Jilin Medical University, Jilin, China

**Keywords:** natural medicinal products, aging, cognitive impairment, learning, memory

Cognition is the acquisition, processing, and storage of external information by the human brain when exposed to external factors, as well as a complex neurophysiological activity including learning, memory, emotion, and thinking. Blocking the basic process of cognitive operation results in cognitive impairment, which mainly manifests as slow reaction and learning, memory decline, etc. (Katzman et al., 1983; Petersen, 2007). The reasons leading to cognitive impairment include diseases such as brain dysplasia, brain trauma, stroke, and cerebral infarction. Some cases are induced by external factors such as frequent drinking, long periods of reduced sleep, abuse of drugs affecting the central nervous system, and exposure to harmful substances. However, most cognitive impairment occurs due to the natural decline of the nervous system caused by aging, which is irreversible functional degeneration and can be delayed only by early interventions in the decline process (Mohammad et al., 2014; Shwe et al., 2020).

Natural medicinal products with high safety and low toxicity are extracted from plants and animals and are suitable for long-term preventive use. Recent studies have demonstrated the good activity of natural medicinal products against aging-related cognitive impairment (Kumar et al., 2008; Li et al., 2012; Du et al., 2015; Turgut et al., 2015; Lee et al., 2017; He et al., 2020; Li et al., 2021). For this research hotspot, we have designed and organized a Research Topic including five articles, all of which are original research focused on introducing the application of natural medicinal products to prevent and ameliorate aging-related cognitive impairment.

This Research Topic includes a variety of natural medicinal products. Wang et al. extracted and separated the ethanol extract (PSCE) from the seed coat of peony, established a mouse cognitive impairment model by administering scopolamine to mice, observed the changes in the cognitive behavior of the model mice, and studied the mechanism of these changes by administering PSCE and its active ingredient suffruticosol B (SB). The results showed that PSCE and SB improved cognitive impairment by regulating cholinergic nerves, antioxidation, and anti-inflammation. Li et al. reported that *Armillaria mellea* polysaccharides improved aging-related learning and memory disorders by regulating the level of oxidative stress kinases and neurotransmitters *in vivo* and promoting the proliferation of neurons in the hippocampus of model mice induced by D-galactose. Zhao et al. reported the effect of a natural medicine compound on cognitive impairment, in which Sagacious Confucius’ Pillow Elixir (SCPE), a Chinese medicine compound, improved cognitive impairment in an aging model in mice. The results showed that SCPE improved cognitive impairment by multiple mechanisms and targets. In addition, this Research Topic includes two articles on the impacts of volatile oil extracts from natural drugs on cognitive impairment, which have been less often reported by previous studies. Guo et al. reported that *Monarda didyma L*. essential oil showed a good ability to improve aging-related cognitive impairment in an aging model in mice and confirmed that the pharmacodynamic effect was related to the Nrf2/MAPK pathway. In another article, Qu et al. observed the effect of *Coreopsis tinctoria* essential oil on cognitive impairment in animal experiments, in which the drugs affected the Nrf2/NF-κB pathway to improve learning and memory disorders in D-galactose-induced mice.

In conclusion, this Research Topic increased our understanding of natural medicinal products to prevent and improve aging-related cognitive impairment, and also provided theoretical evidence for the development and application of natural medicines.
